# Supplementary material for: An explainable language model for antibody specificity prediction using curated influenza hemagglutinin antibodies
Source: bioRxiv. 2023 Sep 14:2023.09.11.557288. Preprint. [Version 1] doi: 10.1101/2023.09.11.557288 (PMC10515799; doi:10.1101/2023.09.11.557288)
Supplement: Supplement 1 [file media-1.pdf]

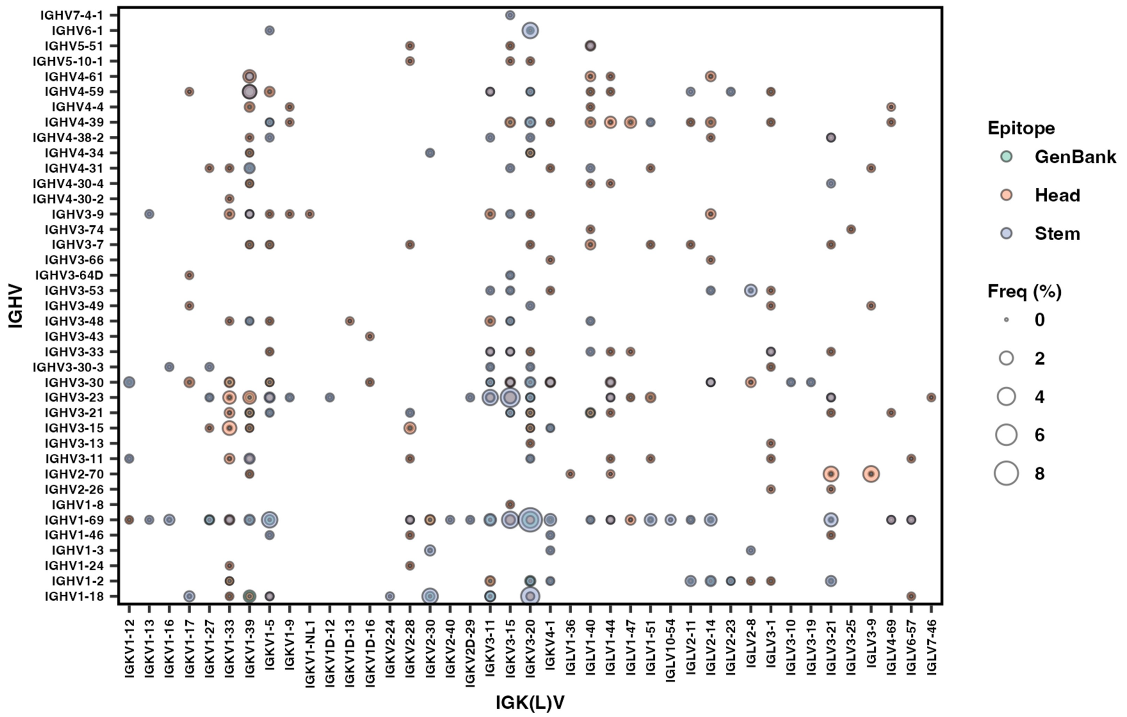

Supplementary Figure 1. Preference of V gene pairings in influenza HA antibodies. The frequencies of different V gene pairings between heavy and light chains are shown for influenza HA antibodies to the head and stem domains. Antibodies from GenBank were also included as a reference. The size of each data point represents the frequency of the corresponding IGHV/IGK(L)V pair within its specificity category. Only those antibodies with both IGHV and IGK(L)V gene information available were included in this analysis.

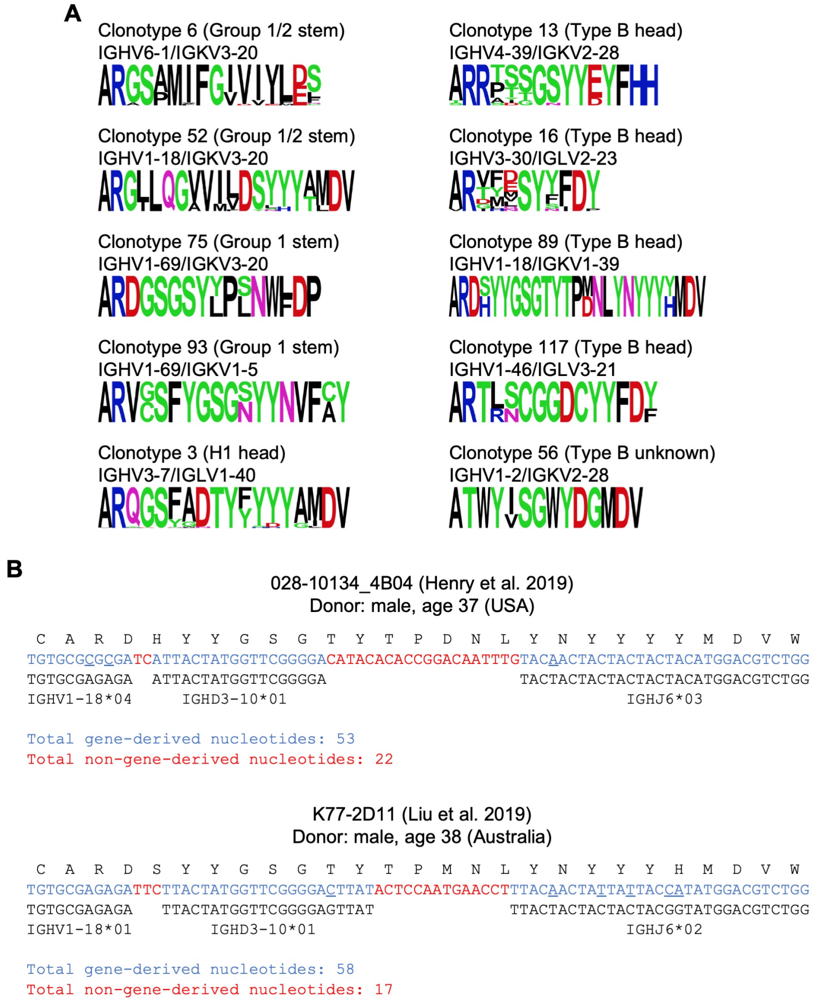

Supplementary Figure 2. Public clonotypes of influenza HA antibodies. (A) Antibodies with the same IGHV/IGK(L)V genes and at least 80% sequence identity in the CDR H3 were defined as a clonotype. A clonotype with antibodies from at least two donors was defined as a public clonotype. A total of 10 public clonotypes were identified. The V gene usage and CDR H3 sequence are shown for each of these 10 public clonotypes. The CDR H3 sequences are shown as a sequence logo, where the height of each letter represents the frequency of the corresponding amino-acid variant (single-letter amino acid code) at the indicated position. This analysis captured many known recurring sequence features in HA stem antibodies, including IGHV6-1 with an [I/V]FG[I/L/V] motif (clonotype 6) [1, 2], VH1-18 with a QxxV motif in CDR H3 (clonotype 52) [3], and IGHV1-69 with a Tyr in the CDR H3 (clonotypes 75 and 93) [4]. The recurring usage of

IGHV3-7/IGLV1-40 among HA head antibodies (clonotype 3) was also noted previously [5, 6]. **(B)** Among the five public clonotypes to influenza type B HA, clonotypes 13, 16, 56, and 117 consisted of antibodies from the same study (**Table S1**) [7, 8]. In contrast, clonotype 89 consisted of antibodies from two different studies [8, 9]. Our dataset contained two antibodies within clonotype 89, namely 028-10134_4B04 and K77-2D11, which were isolated from donors in the US and Australia, respectively [8, 9]. Amino acid and nucleotide sequences of the V-D-J junction are shown for 028-10134_4B04 and K77-2D11. Antibody 028-10134_4B04 was isolated from a 37-year-old male in the US [8], whereas K77-2D11 was isolated from a 38-year-old male in Australia [7]. Putative germline sequences and segments were identified by IgBlast [10] and are indicated. Somatically mutated nucleotides are underlined. Intervening spaces at the V-D and D-J junctions are N-nucleotide additions. Both 028-10134_4B04 and K77-2D11 have a long CDR H3 with 25 amino acids (IMGT numbering), including a YYGSGTY that is largely encoded by IGHD3-10 and a TPxNL motif that is encoded by N-nucleotide addition. While previous studies of recurring sequence features among HA antibodies have mainly focused on influenza type A HA [1-4, 11-15], our results suggest that recurring sequence features among antibodies to influenza type B HA may also be quite common.

A

```
IGHD4-17: TGACTACGGTGACTAC
Frame 1:   *  L  R  *  L
Frame 2:    D  Y  G  D  Y
Frame 3:     T  T  V  T
```

B

W85-1A07 (IGHV3-48/IGLV2-14)

```
 C  A  R  A  H  M  I  Y  G  D  H  V  H  L  N  A  F  D  I  W
TGTGCGAGAGCTCATATGATATACGGTGACCACGTTCATCTGAATGCTTTTGATATTTGG
```

019_10_4A03 (IGHV3-15/IGKV2-28)

```
 C  T  T  D  Y  G  D  Y  L  N  G  G  R  W
TGTACCACTGACTACGGTGACTACCTTAACGGGGGGCGCTGG
```

150055-029_3E03 (IGHV3-23/IGKV1-33)

```
 C  A  K  G  G  Y  G  D  N  G  L  D  V  F  D  I  W
TGTGCGAAAGGAGGATACGGTGACAACGGGTTGGATGTCTTTGATATCTGG
```

SFV009_3F05 (IGHV3-7/IGKV1-5)

```
 C  A  R  A  G  S  Y  G  D  Y  R  P  I  N  N  W  F  D  P  W
TGTGCGAGAGCGGGGAGTTACGGTGACTACAGGCCGATAAACAACTGGTTCGACCCCTGG
```

SFV019_2A02 (IGHV1-18/IGKV1-33)

```
 C  A  R  R  G  D  Y  G  D  Y  R  G  D  A  F  D  I  W
TGTGCGAGACGTGGGGACTACGGTGACTACCGGGGTGATGCATTTGATATCTGG
```

Supplementary Figure 3. YGD motif in IGHD4-17 HA head antibodies. (A) The nucleotide sequence of IGHD4-17 and its amino acid sequences in all three translation frames are shown. **(B)** CDR H3 sequences of representative IGHD4-17 HA head antibodies with different V gene usages. The YGD motif and the IGHD4-17-encoded region are highlighted in red.

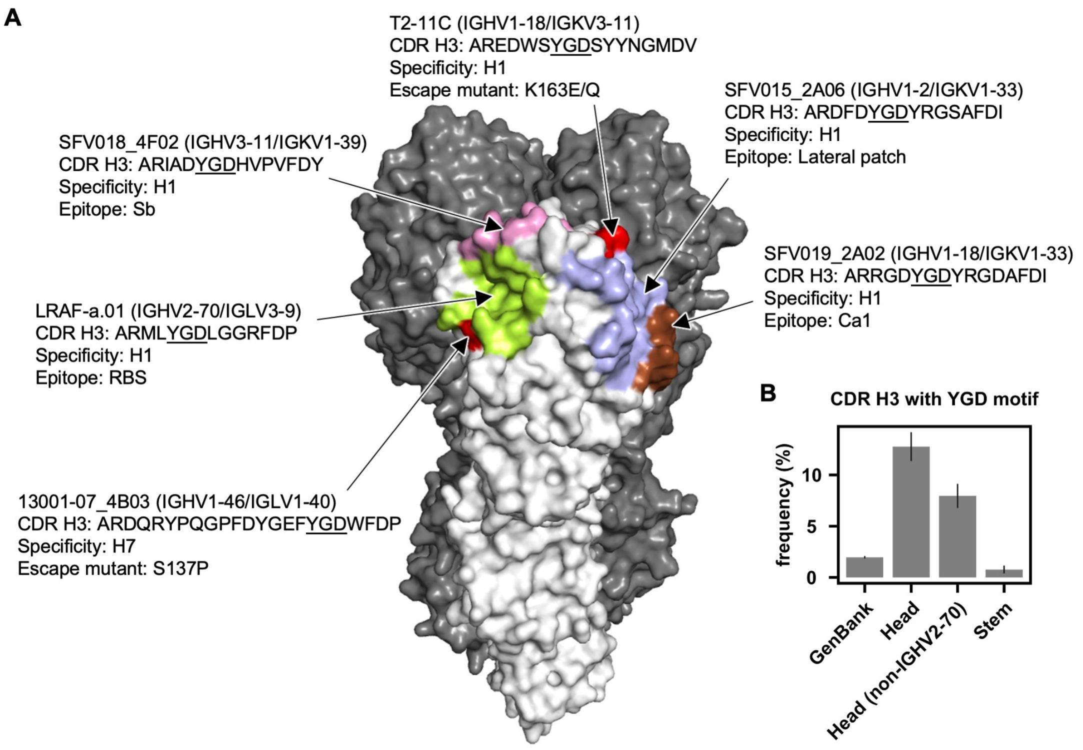

Supplementary Figure 4. Representative IGHD4-17 HA head antibodies with YGD motif. (A) Different epitopes, including the receptor-binding site (RBS, lime), Sb (pink), Ca1 (brown), and lateral patch (blue), are shown on the HA structure (PDB: 3LZG) [16]. Information of representative IGHD4-17 HA head antibodies that target these epitopes is shown. The locations of K163E/Q and S137P, which escape antibodies T2-11C [6] and 13001-07_4B03 [17], respectively, are colored in red. Of note, S137P (H3 numbering) was named as S152P in the original paper [17]. **(B)** Frequency of antibodies with a YGD motif in the CDR H3 among all antibodies from Genbank, HA head antibodies, non-IGHV2-70-encoded HA head antibodies, and HA stem antibodies.

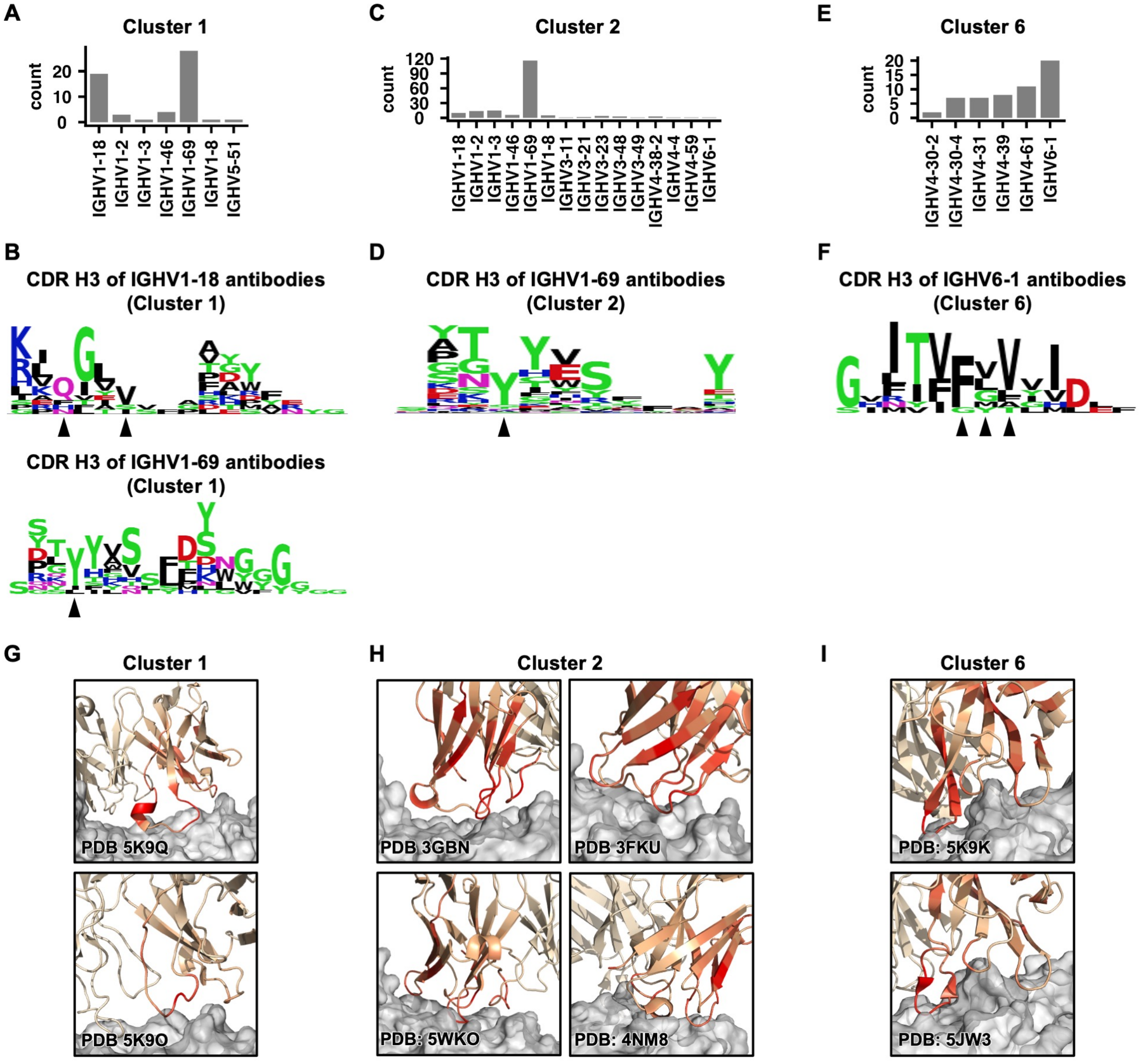

Supplementary Figure 5. Sequence features of clusters 1, 2, and 6 of HA stem antibodies. **(A, C, E)** IGHV gene usages among antibodies in **(A)** cluster 1, **(C)** cluster 2, and **(E)** cluster 6 are shown **(B, D, F)** The saliency score of each CDR H3 residue in **(B)** IGHV1-18 antibodies (top) and IGHV1-69 antibodies (bottom) within cluster 1, **(D)** IGHV1-69 antibodies within cluster 2, and **(F)** IGHV6-1 antibodies within cluster 6 was analyzed. The frequency of each amino acid for residues with a saliency score >0.5 is shown as a sequence logo. Arrows at the bottom indicate the residues of interest, including **(B)** a QxxV motif (top), Y98 (bottom), **(D)** Y98, and **(F)** an FGV motif **(G-I)** Saliency scores are projected onto the structures of **(G)** two antibodies in cluster 1

(PDB 5K9Q and PDB 5K9O [3]), **(H)** four antibodies in cluster 2 (PDB 3GBN [18], PDB 3FKU [19], PDB 5WKO [11], and PDB 4NM8 [20]), and **(I)** two antibodies in cluster 6 (PDB 5K9K [3] and PDB 5JW3 [21]). Color scheme is the same as that in **Figure 4A**.

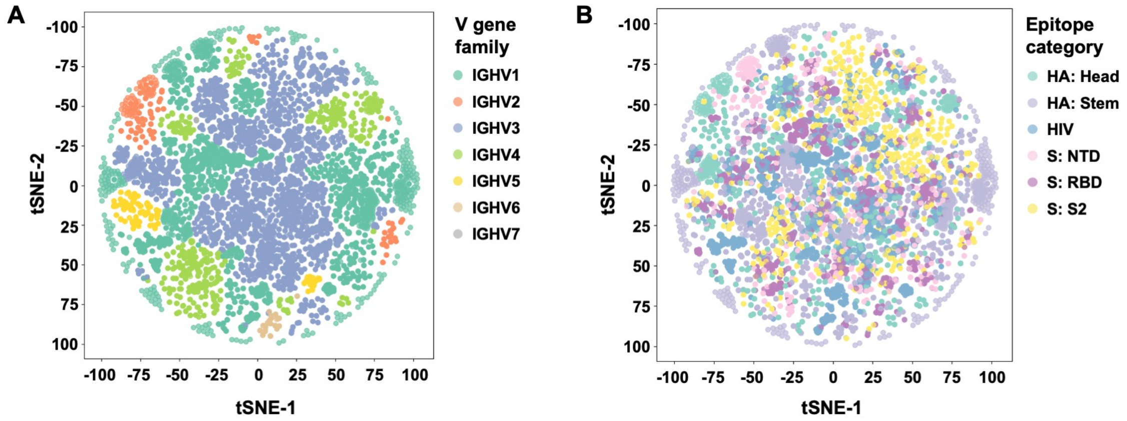

Supplementary Figure 6. t-SNE analysis of the final-layer embeddings of the pre-trained mBLM. The final-layer embeddings of the pre-trained mBLM model (i.e. prior to fine-tuning for specificity prediction) was analyzed by t-SNE (t-distributed Stochastic Neighbor Embedding). Heavy chain sequences in the training set for fine-tuning were used in this analysis. Each datapoint represents one heavy chain sequence. Datapoints are colored by **(A)** V gene families or **(B)** specificity categories.

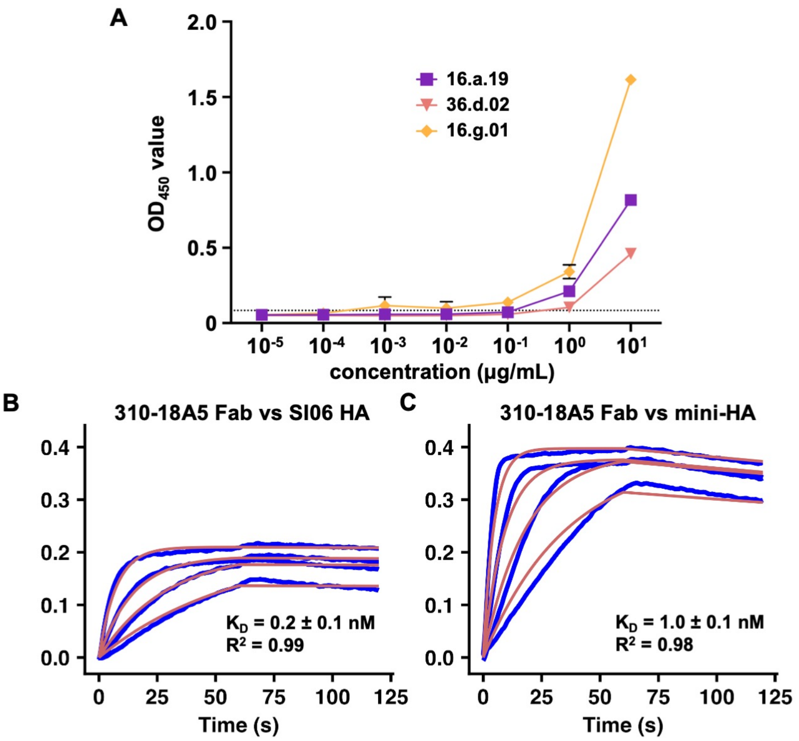

D

Strain	MIC (µg/mL)
H1N1 A/Puerto Rico/8/1934	18.75
H1N1 A/Michigan/45/2015	37.5

Supplementary Figure 7. Binding and neutralization activity of antibodies that were predicted to target HA stem. (A) ELISA was used to test the binding of purified antibodies 16.a.19, 36.d.02, and 16.g.01 at different concentrations to mini-HA. PBS was used as a negative control (dotted line). Data are representative of two independent experiments. **(B-C)** Binding kinetics of 310-18A5 Fab against **(B)** H1N1 A/Solomon Islands/3/2006 (SI06) HA and **(C)** mini-HA were measured by biolayer interferometry (BLI). Y-axis represents the response. Blue lines represent the response curve and red lines represent the 1:1 binding model. Binding kinetics were measured for four concentrations of Fab at 2-fold dilution ranging from 200 nM to 25 nM. Dissociation constant (K_D) and the goodness of model fitting (R^2) are indicated. **(D)** Neutralization activity of 310-18A5 was tested against two H1N1 strains, namely A/Puerto Rico/8/1934 and A/Michigan/45/2015. Minimal inhibitory concentration (MIC) is indicated.

Table S4. Cryo-EM data collection statistics.

	SI06HA-18A5 complex (EMD-41849)
**Data collection and processing**	
Magnification	130,000
Voltage (kV)	200
Electron exposure (e-/Å2)	52.76
Defocus range (μm)	-0.6 to -3
Pixel size (Å)	0.96
Symmetry imposed	C3
Initial particle images (no.)	41,774
Final particle images (no.)	39,446
Map resolution (Å)	4.81
FSC threshold	0.143
Map resolution range (Å)	N/A

Supplementary References

1. Chuang GY, Shen CH, Cheung CS, Gorman J, Creanga A, Joyce MG, et al. Sequence-signature optimization enables improved identification of human HV6-1-derived class antibodies that neutralize diverse influenza A viruses. Front Immunol. 2021;12:662909. Epub 2021/06/18. doi: 10.3389/fimmu.2021.662909. PubMed PMID: 34135892; PubMed Central PMCID: PMCPMC8201785.

2. Wu NC, Andrews SF, Raab JE, O'Connell S, Schramm CA, Ding X, et al. Convergent evolution in breadth of two V_H6-1-encoded influenza antibody clonotypes from a single donor. Cell Host Microbe. 2020;28:434-44. Epub 2020/07/04. doi: 10.1016/j.chom.2020.06.003. PubMed PMID: 32619441.

3. Joyce MG, Wheatley AK, Thomas PV, Chuang GY, Soto C, Bailer RT, et al. Vaccine-induced antibodies that neutralize group 1 and group 2 influenza A viruses. Cell. 2016;166(3):609-23. doi: 10.1016/j.cell.2016.06.043. PubMed PMID: 27453470; PubMed Central PMCID: PMCPMC4978566.

4. Avnir Y, Tallarico AS, Zhu Q, Bennett AS, Connelly G, Sheehan J, et al. Molecular signatures of hemagglutinin stem-directed heterosubtypic human neutralizing antibodies against influenza A viruses. PLoS Pathog. 2014;10(5):e1004103. doi: 10.1371/journal.ppat.1004103. PubMed PMID: 24788925; PubMed Central PMCID: PMCPMC4006906.

5. Krause JC, Tsibane T, Tumpey TM, Huffman CJ, Briney BS, Smith SA, et al. Epitope-specific human influenza antibody repertoires diversify by B cell intraclonal sequence divergence and interclonal convergence. J Immunol. 2011;187(7):3704-11. Epub 2011/09/02. doi: 10.4049/jimmunol.1101823. PubMed PMID: 21880983; PubMed Central PMCID: PMCPMC3178754.

6. Huang KY, Rijal P, Schimanski L, Powell TJ, Lin TY, McCauley JW, et al. Focused antibody response to influenza linked to antigenic drift. J Clin Invest. 2015;125(7):2631-45. doi: 10.1172/JCI81104. PubMed PMID: 26011643; PubMed Central PMCID: PMCPMC4613558.

7. Liu Y, Tan HX, Koutsakos M, Jegaskanda S, Esterbauer R, Tilmanis D, et al. Cross-lineage protection by human antibodies binding the influenza B hemagglutinin. Nat Commun. 2019;10(1):324. Epub 2019/01/20. doi: 10.1038/s41467-018-08165-y. PubMed PMID: 30659197; PubMed Central PMCID: PMCPMC6338745 and co-funded by Sanofi Pasteur, which is the manufacturer of influenza vaccines (FluQuadri) received by some, but not all, of the participants of this study. The remaining authors declare no other competing interests.

8. Henry C, Zheng NY, Huang M, Cabanov A, Rojas KT, Kaur K, et al. Influenza virus vaccination elicits poorly adapted B cell responses in elderly individuals. Cell Host Microbe. 2019;25(3):357-66.e6. Epub 2019/02/24. doi: 10.1016/j.chom.2019.01.002. PubMed PMID: 30795982.

9. Thomson CA, Wang Y, Jackson LM, Olson M, Wang W, Liavonchanka A, et al. Pandemic H1N1 influenza infection and vaccination in humans induces cross-protective antibodies that target the hemagglutinin stem. Front Immunol. 2012;3:87. doi:

10.3389/fimmu.2012.00087. PubMed PMID: 22586427; PubMed Central PMCID: PMCPMC3347682.

10. Ye J, Ma N, Madden TL, Ostell JM. IgBLAST: an immunoglobulin variable domain sequence analysis tool. Nucleic Acids Res. 2013;41(Web Server issue):W34-W40. doi: 10.1093/nar/gkt382. PubMed PMID: 23671333; PubMed Central PMCID: PMCPMC3692102.

11. Lang S, Xie J, Zhu X, Wu NC, Lerner RA, Wilson IA. Antibody 27F3 broadly targets influenza A group 1 and 2 hemagglutinins through a further variation in V_H1-69 antibody orientation on the HA stem. Cell Rep. 2017;20(12):2935-43. doi: 10.1016/j.celrep.2017.08.084. PubMed PMID: 28930686.

12. Wu NC, Yamayoshi S, Ito M, Uraki R, Kawaoka Y, Wilson IA. Recurring and adaptable binding motifs in broadly neutralizing antibodies to influenza virus are encoded on the D3-9 segment of the Ig gene. Cell Host Microbe. 2018;24(4):569-78.e4. doi: 10.1016/j.chom.2018.09.010. PubMed PMID: 30308159.

13. Cheung CS, Fruehwirth A, Paparoditis PCG, Shen CH, Foglierini M, Joyce MG, et al. Identification and structure of a multidonor class of head-directed influenza-neutralizing antibodies reveal the mechanism for its recurrent elicitation. Cell Rep. 2020;32(9):108088. Epub 2020/09/03. doi: 10.1016/j.celrep.2020.108088. PubMed PMID: 32877670.

14. Crowe JE, Jr. Influenza virus-specific human antibody repertoire studies. J Immunol. 2019;202(2):368-73. Epub 2019/01/09. doi: 10.4049/jimmunol.1801459. PubMed PMID: 30617118; PubMed Central PMCID: PMCPMC6327975.

15. Andrews SF, Graham BS, Mascola JR, McDermott AB. Is it possible to develop a “universal" influenza virus vaccine? immunogenetic considerations underlying B-cell biology in the development of a pan-subtype influenza A vaccine targeting the hemagglutinin stem. Cold Spring Harb Perspect Biol. 2018;10(7):a029413. Epub 2017/07/01. doi: 10.1101/cshperspect.a029413. PubMed PMID: 28663207; PubMed Central PMCID: PMCPMC6028068.

16. Xu R, Ekiert DC, Krause JC, Hai R, Crowe JE, Jr., Wilson IA. Structural basis of preexisting immunity to the 2009 H1N1 pandemic influenza virus. Science. 2010;328(5976):357-60. doi: 10.1126/science.1186430. PubMed PMID: 20339031; PubMed Central PMCID: PMCPMC2897825.

17. Henry Dunand CJ, Leon PE, Huang M, Choi A, Chromikova V, Ho IY, et al. Both neutralizing and non-neutralizing human H7N9 influenza vaccine-induced monoclonal antibodies confer protection. Cell Host Microbe. 2016;19(6):800-13. Epub 2016/06/10. doi: 10.1016/j.chom.2016.05.014. PubMed PMID: 27281570; PubMed Central PMCID: PMCPMC4901526.

18. Ekiert DC, Bhabha G, Elsliger MA, Friesen RH, Jongeneelen M, Throsby M, et al. Antibody recognition of a highly conserved influenza virus epitope. Science. 2009;324(5924):246-51. doi: 10.1126/science.1171491. PubMed PMID: 19251591; PubMed Central PMCID: PMCPMC2758658.

19. Sui J, Hwang WC, Perez S, Wei G, Aird D, Chen LM, et al. Structural and functional bases for broad-spectrum neutralization of avian and human influenza A viruses. Nat Struct Mol Biol. 2009;16(3):265-73. doi: 10.1038/nsmb.1566. PubMed PMID: 19234466; PubMed Central PMCID: PMCPMC2692245.

20. Friesen RH, Lee PS, Stoop EJ, Hoffman RM, Ekiert DC, Bhabha G, et al. A common solution to group 2 influenza virus neutralization. Proc Natl Acad Sci U S A. 2014;111(1):445-50. doi: 10.1073/pnas.1319058110. PubMed PMID: 24335589; PubMed Central PMCID: PMCPMC3890827.

21. Kallewaard NL, Corti D, Collins PJ, Neu U, McAuliffe JM, Benjamin E, et al. Structure and function analysis of an antibody recognizing all influenza A subtypes. Cell. 2016;166(3):596-608. doi: 10.1016/j.cell.2016.05.073. PubMed PMID: 27453466; PubMed Central PMCID: PMCPMC4967455.
